# Factors associated with hospitalization after self-poisoning in France: special focus on the impact of alcohol use disorder

**DOI:** 10.1186/s12888-018-1854-0

**Published:** 2018-09-06

**Authors:** Juliette Salles, Julie Calonge, Nicolas Franchitto, Emmanuelle Bougon, Laurent Schmitt

**Affiliations:** 1 Université de Toulouse III, F-31000 Toulouse, France; 2 CHU Toulouse, Service de Psychiatrie eSt Psychologie, Psychiatrie, F-31000 Toulouse, France; 3 CHU Toulouse, Service d’addictologie clinique, urgences réanimation médecine, F-31000 Toulouse, France

**Keywords:** Suicide, Epidemiology, Hospitalization, Alcohol use disorder

## Abstract

**Background:**

Previous studies have identified factors associated with admission to hospital after suicide spectrum behaviors. In this study, we aim to identify specific factors associated with psychiatric hospitalization after self-poisoning. Given earlier findings suggesting that alcohol use disorder is not associated with hospital admission, we also aim to consider its impact, as well as blood alcohol concentrations, on hospitalization decisions after a suicide attempt.

**Methods:**

We studied the association between demographic features, suicide intent, psychiatric characteristics and admission to hospital in self-poisoning patients in an emergency department in France.

**Results:**

Suicide intent, a past history of suicide attempts, bipolar disorder and depression were associated with psychiatric hospital admissions. Despite alcohol use disorder being known to be associated with a suicide risk, it was not linked with psychiatric hospitalization. A positive blood alcohol concentration in the emergency department likewise had no association with admission to a psychiatric ward for inpatient care.

**Conclusions:**

Our findings were similar to those reported for other suicide spectrum behaviors. Alcohol use disorder was not associated with admission for inpatient psychiatric care, whereas depression clearly was. The cause of this discrepancy must be determined in future research.

## Background

Suicide is a major global public health concern and in Europe represents 1.5% of all deaths [1]. The causes of suicide are multiple and complex and can be linked to psychological, environmental and cultural factors. Among the psychological factors, mental illnesses such as mood disorders, schizophrenia, anxiety disorders, post-traumatic stress and substance abuse [2–6] play an important role and must be regarded as specific suicide risk factors.

In terms of suicide care, 80% of patients receiving medical help after a suicide attempt are admitted to the emergency ward. In France, every patient hospitalized for an attempted suicide benefits from a psychiatric assessment [7]. This assessment is an opportunity to evaluate the suicide risk, initiate treatment if required and prevent future self-harm. The assessment of suicide spectrum behaviors in the emergency department is a key factor in preventing suicide [8]. The suicidal patient may be discharged or hospitalized based on the assessment. There are many guidelines on how suicide assessments should be conducted, but none are universally accepted [9–11]. The referral of patients for hospitalization or discharge is a crucial clinical decision, and most guidelines recommend that direct discharge from the emergency department should be considered if an after-care plan can be arranged before the patient leaves hospital. In France, attempted suicide by self-poisoning represents 81.7% of all suicide attempts. Overall, 66% of self-poisoned patients are women versus 63% for the entire population of suicide attempters versus 45% for other methods. The frequency of self-poisoning versus other suicide methods decreases with age [12]. Compared with other methods, previous studies have found that patients who self-poison are less often psychotic and more often have an anxiety or affective disorder than those admitted following the use of violent methods such as hanging, cutting, jumping, and the use of firearms. However, no differences in demographic characteristics (age, marital status, living conditions, educational status and employment status) and a past history of suicide attempts have been identified [13].

Hospitalization is recommended if there is an imminent risk of suicide. Admission policies for suicide treatment have been associated with clinical and psychological factors such as older age, male gender, poor physical health, psychosis, high suicidal intent, a plan to use a lethal method, previous psychiatric hospitalization, a past history of suicide attempts and a low expectation of being found after an attempt [8, 14]. Hospitalization is generally indicated if the attempt was violent or premeditated, precautions were taken to avoid rescue, distress is increased, the patient regrets surviving, the patient has limited social support, there is current impulsive behavior or a refusal of help is evident, and the patient has a change in mental status with a metabolic, toxic, infectious or other etiology requiring further workup in a structured setting. However, when considering hospitalization, the risk of suicide is not the only factor to take into account, as admission to hospital could reinforce dependency and regressive behaviors. Psychiatric inpatients and their relatives may also have unrealistic expectations and hospitalization can be associated with disillusionment and may contribute to feelings of hopelessness. Moreover, in cases of personality disorder, it may lead to a vicious circle of intensifying suicidal thoughts that require more restrictive care [15].

Within psychiatric disorders, alcohol use disorder (AUD) is particularly associated with an increased suicide mortality rate, which is six times higher than in the general population [16]. A literature review put the suicide risk over a lifetime at 3.4% in AUD populations in the USA [17], while a meta-analysis found a strong association between suicide attempts and alcohol consumption (OR = 3.1) [18].

Despite the impact that acute alcohol consumption has on decision making, AUD appears to not be related to clinicians’ decisions to hospitalize, even though the association between AUD and suicide is largely documented. Indeed, one study [19] specifically addressed the issue of clinical decision-making with respect to hospitalization in a population of patients demonstrating suicide spectrum behaviors, including suicidal ideation, non-suicidal injuries and suicide attempts. This research found that AUD was not associated with admission for inpatient psychiatric care, whereas schizophrenia and depression were. The authors hypothesized that clinicians’ decisions about hospitalization could be more related to drug or alcohol consumption associated with suicide attempts than to AUD [20].

Previous data on factors associated with hospital admissions are available for suicide spectrum behaviors, especially in Spain [8, 19]. However, there is a difference between countries concerning care management, which is why we sought to replicate those results in France. In particular, we aimed to identify factors associated with psychiatric hospitalization, especially in a population of self-poisoning suicide attempters. Regarding previous results, we also aimed to pay specific attention to the impact of AUD and blood alcohol concentrations (BAC) on clinicians’ decisions concerning hospitalization.

## Methods

### Participants

The study took place in Toulouse University Hospital, which is located in the south of France. This hospital benefits from an emergency room that is specifically set up to deal with self-poisoning patients. This ward is a medical department directed by an emergency team in which a psychiatric liaison team ensures that appropriate psychiatric assessments are carried out. This team consists of a nurse and a psychiatrist working 7 days a week, 24 h a day. All patients who attempt suicide by self-poisoning are referred to this unit.

The WHO definition of a suicide attempt was used, namely: any “act with a non-fatal outcome, in which an individual deliberately initiates a non-habitual behavior that without intervention from others will cause self-harm or deliberately ingests a substance in excess of the prescribed or generally recognized therapeutic dosage” [21].

**Fig. 1:**
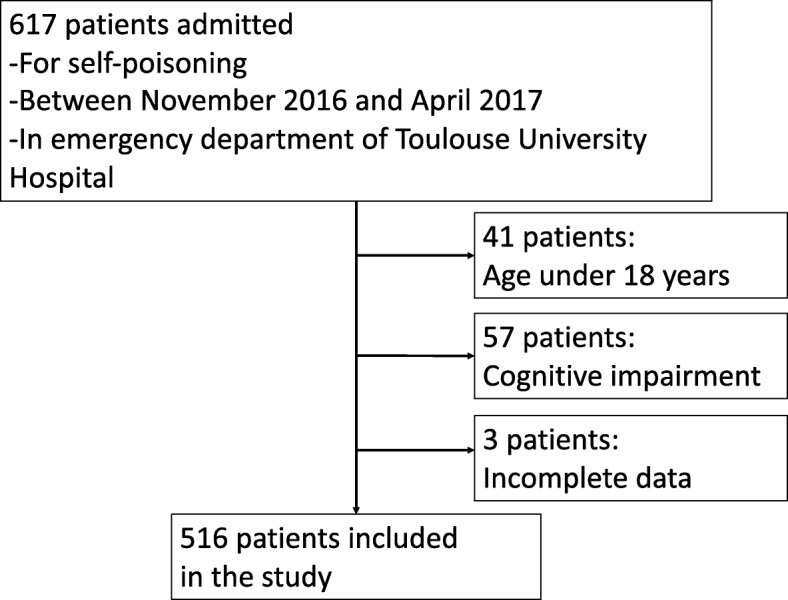
Flow chart

This article is based on data initially collected between November 2016 and April 2017 for an Intent of Suicide and Alcohol (ISA) study. Patients were excluded if they were under 18 years old, because of the legal liability for research participation. Patients with a diagnosis of mild or severe mental disability and mild or severe neurocognitive impairment were also excluded to ensure the quality of responses. We used administrative data to compare included attempters versus non-included attempters. During the relevant period, 98 patients did not meet the inclusion criteria. In total, 519 patients were recruited, but three were excluded because of incomplete data (Fig. 1).

### Measurements

The data collected included patients’ sociodemographic and clinical features, personal and familial history of suicide attempts and type of follow-up (inpatient or outpatient care).

Sociodemographic characteristics consisted of age, gender, living status (alone or not) and employment status.

Clinical features comprised psychiatric diagnoses according to the DSM IV criteria, which were assessed by referring to the Mini International Neuropsychiatry Interview (MINI) subset (A-I) [22].

Data on prior mental health-care included previous psychiatric follow-up by a psychiatrist, a history of suicide attempts and a personality disorder diagnosis. This data was collected from the information in patient assessments or the emergency database concerning previous medical involvement.

Data concerning suicide attempts included information obtained from the Intent of Suicide Assessment, which is based on the Pierce Scale [23]. The Pierce Scale is a 12-item questionnaire designed to assess the severity of suicide intent associated with an episode of self-harm. Each item scores 0–3, giving a total score of 0–25. The questionnaire is divided into three sections: the first six items constitute the “circumstances” section and are concerned with the objective circumstances of the act of self-harm; the following four items constitute the “self-report” section and are based on patients’ own reconstructions of their feelings and thoughts at the time of the act; finally the remaining two items constitute the “risk” section and are concerned with the medical risk of self-harm relative to the lethality of the suicide method.

Blood samples were taken systematically at the time of admission to the emergency unit in order to determine blood alcohol concentrations (BACs).

Referrals were separated into two pathways:

-Outpatient psychiatric care: this consisted of an at-home psychiatric appointment with a primary psychiatrist, information to make an appointment with a psychiatrist in public medical psychological centers (CMPs) or an appointment with a psychiatrist in a brief therapy center (CTB).

-Inpatient psychiatric care: this consisted of either voluntary or forced hospitalization in a psychiatric department.

The psychiatric assessments were performed in an emergency ward that enabled all patients to be evaluated, including those who were severely intoxicated. Evaluations were performed after cognitive assessments and negative BACs were obtained to ensure that patients were capable of being assessed.

Referrals were systematically determined by a psychiatrist during a psychiatric assessment.

We also collected data relating to repeat suicide attempts at 1 year from the administrative database of Toulouse University’s emergency department.

### Data analysis

We compared sociodemographic characteristics and clinical features according to the referral after the psychiatric assessment. Quantitative variables are described using the mean (+/− standard deviation) and/or median (+/− interquartile range) according to their distributions.

The Shapiro-Wilk test was performed to evaluate the normality distribution of the data. In cases of non-normal data, the Wilcoxon non-parametric test was applied; the Student T test was used for normal data. Associations between qualitative variables were tested using the chi-squared or the Fischer test (when expected values were less than five). Statistical significance was set at *p* < 0.05. Multinomial logistic regression was used to assess the association between clinical features (past history of suicide, depression, schizophrenia, bipolar disorder, AUD, personality disorder, suicide intent, BACs), sociodemographic characteristics (age, gender, living status: alone or not, living status: housed or not, and professional status: employed or not), and the outcome of being referred for psychiatric hospitalization or psychiatric outpatient care. We used a logistic analysis to identify variables independently associated with the referrals [24]. We then performed backward stepwise analyses to achieve our final model. Statistical significance was set at *p* < 0.05. We tested the interactions between all the variables included in the final model.

The statistical analyses were performed using the R-Studio software, version 14R (StataCorp LP, College Station, TX, USA).

## Results

### Sample description

A total of 165 men (31.9%) and 351 women (68.1%) were included in the sample. The mean age was 40.5 (SD = 14.8) and the median age 42 (IQ = 27–51). Overall, during self-poisoning, 38.58% of patients ingested two medications, 10.85% ingested three and 1.16% ingested more than three. The most common medications used were benzodiazepines, with 85% of patients ingesting at least one of these. Paracetamol accounted for 8.91% of the ingested drugs and opioids 3.68%. The characteristics of the overall sample are described in Table 1.

**Table 1:** Descriptive characteristics of the study population

	N	%
Age		
18-39 years	233	45.16
40-59 years	242	46.90
more than 60 years	41	7.95
Gender		
Men	165	31.98
Women	351	68.02
Number of medication used		
One	516	100
Two	199	38.56
Three	56	10.85
More than three	6	1.16
Type of drug used		
Benzodiazepine	439	85
Paracetamol	46	8.91
Non-steroidal anti-inflammatory Drugs	25	4.84
Neuroleptic	46	8.91
Antidepressant	26	5.03
Opioids	19	3.68
Others	35	6.78
Not known	10	1.93
Past history of suicide	308	59.69
Family past history of suicide	92	17.83
Psychiatric disorder	351	68.02
Depression	170	32.95
Schizophrenia	18	3.49
Bipolar disorder	59	11.43
Alcohol use disorder (AUD)	155	30.04
AUD and depression	54	34.83
AUD and bipolar disorder	13	8.38
AUD and schizophrenia	4	2.58
AUD and personality disorder	65	41.93
Borderline	23	14.83
Dependent	31	20.00
Avoiding	2	1.29
Histrionic	1	0.64
Narcissistic	1	0.64
Obsessive	1	0.64
Antisocial	6	3.87
Personality disorder	178	34.50
Borderline	104	20.16
Dependent	45	8.72
Avoiding	1	0.19
Histrionic	6	1.16
Narcissistic	3	0.58
Obsessive	2	0.39
Antisocial	17	3.29
Single	179	34.69
Employed	198	38.37
Housing	474	91.86

Most of the patients did not live alone (65.1%), 38.0% were employed and 92.0% had a home. More than half of the patients had a history of suicide attempts (59.7%) and 17.8% had a family history of suicide. In our sample, according to the DSM IV criteria, the AUD prevalence was 30.0%, the mood disorder prevalence 44.0%, including 33.0% with depression and 11.0% with bipolar disorder, and the schizophrenia prevalence was 3%. Thirty-four percent of patients had a previous diagnosis of a personality disorder, mostly borderline personality (58.2%).

After psychiatric assessment, 32.9% of patients were referred for inpatient psychiatric care and 67.1% to outpatient care. Compulsory inpatient admissions comprised 9% of the total admissions.

The repeat suicide attempt rate within the one-year follow-up period was 25.51%: 24.69% for inpatient care and 28.10% for outpatient care. Patients suffering from AUD had a higher rate of repetition of 38%, versus 24% in the non-AUD group. In patients with personality disorders, the repetition rate in this sub-group was similar between those with or without AUD: respectively 34.53% and 36.0%. When they were referred for inpatient care, patients with a personality disorder and AUD made fewer repeat suicide attempts than those without AUD: respectively 16.66% versus 42.10%. Adjusting for the Pierce score and sociodemographic factors, we found that in the non-AUD group inpatient orientation reduced the rate of repeat attempts by 2% at 1 year compared to outpatient care. Moreover, in the AUD group, inpatient care reduced the rate of repeat attempts by 5% at 1 year compared to outpatient care.

### Factors associated with referral for psychiatric hospitalization

Table 2 presents the characteristics associated with inpatient psychiatric care determined by univariate and multivariate analyses. We chose to include variables previously associated with a suicide risk in the model.

**Table 2:** Characteristics associated with psychiatric hospitalization

	Hospitalization				
	Univariate comparison			Multivariate logistic regression	
	Yes(*n*=162)	No(*n*=354)	p	OR [95%IC]	p
Sociodemographics					
Age (years)	43.9	38.8	**<0.001**	1.01 [0.99-1.02]	0.12
Gender Men (*n*=165)	48 (29.6)	117 (33)	0.65	0.91 [0.55-1.53]	0.74
Living status (*n*=474) Housing or not	155 (95.6)	319 (90.1)	0.69	1.16[0.45-3.38]	0.76
Living status (*n*=179) Alone or not	58 (35.8)	121 (34.1)	0.85	1.23 [0.77-1.98]	0.37
Professional status (*n*=198) Employee or not	68 (41.9)	130 (36.7)	0.49	1.25 [0.78-2.01]	0.34
Psychopathology and care characteristics					
Depression (*n*=170)	89 (54.9)	81 (22.8)	**<0.001**	3.6 [2.18-6.05]	**<0.001**
Schizophrenia (*n*=18)	5 (3.1)	13 (3.7)	0.95	1.63 [0.43-5.4]	0.43
Bipolar disorder (*n*=59)	23 (14.2)	36 (10.1)	0.29	3.16 [1.57-6.33]	**<0.001**
Alcohol use disorder(*n*=155)	43 (26.5)	112 (31.6)	0.45	0.87 [0.49-1.54]	0.65
Personality disorder(*n*=178)	29 (17.9)	150 (42.3)	**<0.001**	1.03 [0.62-1.71]	0.89
Suicidal intentionnality (Pierce Score)	9.8 (5.7)	4.5 (4.4)	**<0.001**	1.18 [1.13-1.24]	**<0.001**
Past history of suicide (*n*=308)	107 (66.1)	201 (59.3)	0.34	1.53 [0.95-2.47]	**0.07**
Blood alcohol concentration (BAC) mean (SD)	0.63 (0.93)	0.62 (0.92)	0.11	0.87 [0.64-1.18]	0.39

In the univariate analysis, age (*p* ≤ 0.001) and depression (*p* ≤ 0.001) were significantly associated with psychiatric hospitalization, whereas personality disorder (*p* ≤ 0.001) was associated with outpatient care.

In the multivariate analysis, compared to those who had no depression, those who were depressed were more often hospitalized in the psychiatric department (OR = 3.6, 95% CI = 2.18–6.05). Bipolar disorder (OR = 3.16; 1.57–6.33), a history of suicide attempts (OR = 1.53; 0.95–2.47) and suicide intent (OR = 1.18; 0.13–1.24) were also associated with a higher rate of hospitalization.

The association of a personality disorder or schizophrenia with hospitalization was not significant.

Although AUD was significantly related to a suicide risk, patients suffering from the condition did not have a higher likelihood of being referred to inpatient care (OR = 0.87; 0.49–1.54) (*p* = 0.65). BACs were also not associated with a clinician’s decision to hospitalize a patient (OR = 0.87; 0.64–1.18), (*p* = 0.39) (Fig. 1).

**Fig. 2:**
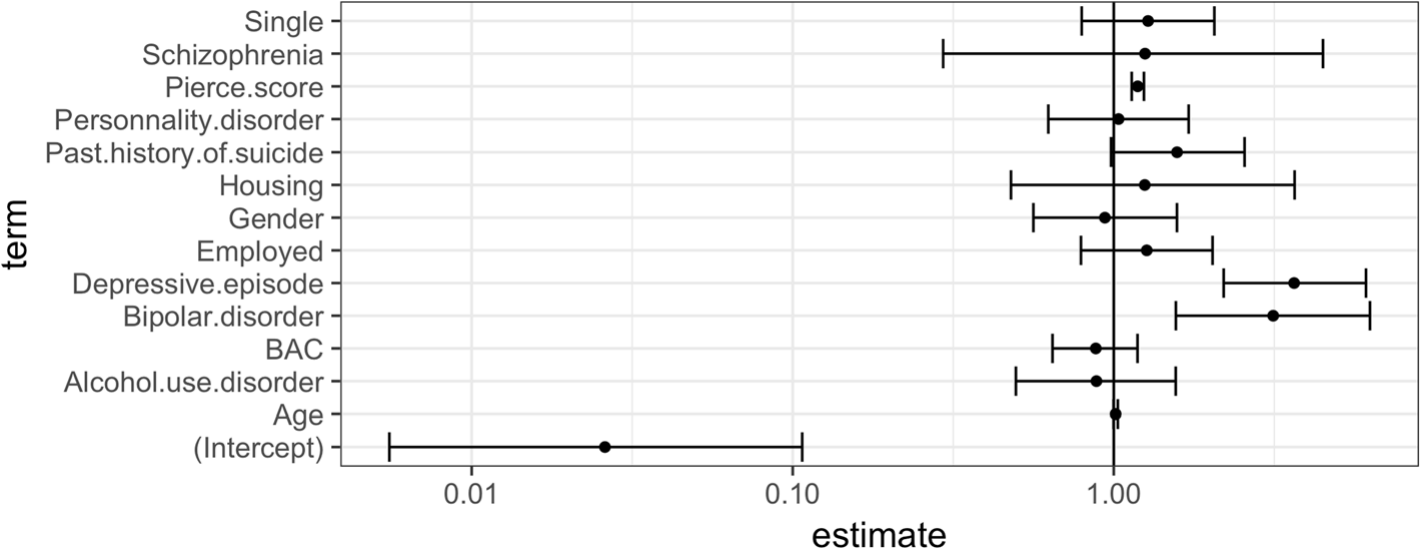
Factors associated with hospitalization. The figure represents the OR as an estimate. On the x-axis part of the figure, the left of the y-axis represents outpatient care and the right part inpatient care

As the presence of a personality disorder often affected the referral decision, we performed a regression analysis on patients that excluded this as a variable. The results were similar to those observed in the overall sample. Indeed, we noted that depressive episodes (OR = 3.58, 95% CI = 1.93–6.80) (*p* < 0.001), bipolar disorder (OR = 2.36, 95% CI = 1.02–5.44) (*p* = 0.04), the Pierce score (OR = 1.17, 95% CI = 1.11–1.23) (*p* < 0.001) and housing status (OR = 3.21, 95% CI = 1.87–16.97) (*p* < 0.001) were associated with inpatient referrals Fig. 2.

## Discussion

The main finding of our study was that AUD was not associated with psychiatric admissions. This also underlined a discrepancy in the consideration of AUD and other psychiatric diagnoses such as depression and bipolar disorders, which were associated with more inpatient referrals.

### Admission rate

The total admission rate of 32.9% in our sample is higher than that previously described in Spain (26.8%) [19] or Finland (24%) [25], but is lower than the rate in Switzerland, England or the WHO/Euro Multicentre Study on Parasuicide, in which almost half of patients were admitted for inpatient care after a suicide attempt [9, 26, 27].Those differences in hospitalization rates could be explained by differences in patient characteristics, care organizations and clinical practices.

### Factors associated with psychiatric hospitalization

Depression was the main predictive factor of psychiatric inpatient care. Depression is also the most common psychiatric disorder in suicide attempters [28, 29]. This was also the case in our study, with it accounting for 48% of all psychiatric diagnoses. Bipolar disorder was independently associated with increased hospitalization. This is unsurprising, as it is known to be associated with suicide attempts or death by suicide [30]. Contrary to a previous study [19], schizophrenia and personality disorder were not associated with psychiatric inpatient care in our sample. In relation to the former, this could be due to the small representation (only 3% of patients) of this diagnosis in our study.

In our sample, 20.16% of patients suffered from a borderline personality disorder. This is relevant because, personality disorders, especially borderline personality, are commonly observed among individuals who attempt suicide. We found no association between personality disorder and inpatient referral. This could be explained by the fact that the role of hospitalization after suicide attempts has generated controversy. One group advocates that hospitals keep patients safe and are appropriate for the treatment of co-morbidities, diagnosis clarifications and simplifying prescription medications [31, 32]. Others point out that minimal evidence exists concerning the efficacy of hospitalization for chronic suicidality. Moreover, hospitalization can result in negative consequences for patients with a borderline personality, including behavioral regression [33].

In terms of the data regarding suicide characteristics, and in parallel with the results of [8], we found that a previous suicide attempt was associated with impatient care, as was suicidal intent.

Surprisingly, we found no association with sociodemographic characteristics, despite previous studies finding a link between male gender and psychiatric inpatient care. One explanation could be a recruitment bias among suicide attempters, as we only recruited the self-poisoning population, which comprised a large majority of women (68%). However, the careful evaluation of other important clinical variables (suicide intent, psychiatric disorder and sociodemographic) has tempered the gender effect.

BACs were not associated with inpatient orientation, which is probably explained by the fact that the patient assessment is only performed when the estimated BAC is below 0.05 g/L.

AUD is also a well-recognized suicide risk factor, but was not associated with psychiatric inpatient care decisions in our sample. We also found no association between BACs and the decision to hospitalize.

This finding underlines the fact that, in our center, AUD is considered differently to depression in clinician decisions, whereas previous studies found a similar suicidal risk increase with a depression or AUD diagnosis (respectively OR = 2.2 and OR = 2.4) [34]. Since the decision to treat a suicide attempter as an outpatient is often due to these diagnoses, we performed an analysis on the sample excluding personality disorders and achieved a similar result to that for the entire population. This tends to confirm that AUD is considered differently to depression, even if patients do not suffer from a personality disorder.

Comparing patients with depression and co-morbid AUD, we found that they were less often referred for inpatient care than non-AUD patients (46.4% versus 53.5%).

Moreover, care referrals depended on the level of the risk of repetition, and AUD is related to multiple suicide attempts [35, 36] and rapid reattempts, as 50% of patients plan their suicide attempt less than 1 week before it takes place [37–39], although unplanned suicide attempts can still be deadly [40]. In addition, alcohol intoxication could promote a suicide attempt because of its disinhibitory effect [38, 39], and patients with AUD are at risk of alcohol consumption, including after a suicide attempt.

A longer period of follow-up is the most protective factor when it comes to preventing another suicide attempt [41], which is why, in practice, a psychiatric evaluation needs to assess the effectiveness of outpatient care. Inpatient care after a suicide attempt does not lead to any reduction in the long-term suicide rate compared to outpatient care; in fact, the main protective factors consist of a care program independent of inpatient or outpatient modalities [28, 42, 43]. However, it has been shown that patients suffering from substance abuse, including AUD, have difficulty engaging in outpatient care [44, 45]. This could therefore mean that inpatient care should be specifically considered during a suicide crisis in order to place the patient at a distance from alcohol, thereby reducing impulsivity, improving mood and promoting follow-up observance [46, 47].

In our database, we found that patients with AUD had a higher risk of repetition than those without AUD. We also found that outpatient care reduced the repetition rate at 1 year in the AUD versus the non-AUD population. This result also led to consideration of inpatient referrals in this group. However, the study was not designed to evaluate repetition rates and we therefore only report retrospective descriptive data concerning the evolution at 1 year. This finding must therefore be confirmed by future studies that specifically focus on this issue.

The discrepancy between the impact of AUD and depression on clinician decisions also needs to be addressed. It could partly be explained by the stigmatization of patients with AUD. Indeed, AUD is the most judged behavior compared with other health conditions such as obesity, depression or schizophrenia [48]. Previous studies reported in the literature demonstrated that AUD was less commonly regarded as a mental illness than depression or schizophrenia [49]. Nevertheless, a previous study showed that holding a conception of AUD as a mental illness increased treatment access [50]. In addition, a study including internal medicine residents found a lower regard for AUD patients than those with other common conditions [51]. Further, previous data suggest that patients hospitalized for suicide risk who are judged to have a risk related to alcohol intoxication are discharged sooner than those who are not perceived to have a substance-related risk [52]. This knowledge led to consideration of whether AUD is stigmatized and its implications for a clinician’s decision-making, including by psychiatrists, when it comes to treating suicide attempters. In our study, it is possible to hypothesize that different views of AUD and other psychiatric disorders could partly lead to care discrimination through a clinician’s decision not to associate AUD with admission to hospital.

A limitation of this study is that we did not take the types of care for other addictions into account. Other addictions are reported on less, suggesting that practitioners do not consider them when deciding the type of care that a patient requires. Another limitation is that this was a single center study and, despite this center evaluating patients using international guidelines, our observations could not be generalized to all patients seen in France after a self-poisoning suicide attempt. Despite self-poisoning accounting for 82% of suicide attempts in France, this population is very specific. For example, other groups, such as those who attempt suicide by hanging or the use of firearms, are mostly represented by men and are suicide methods associated with greater lethality. Moreover, previous studies have referred to heterogeneity in a population with AUD that is explained by different settings and different populations, leading us to emphasize that our findings are limited to patients with AUD who attempted suicide by self-poisoning [33]. Another limitation is the study’s cross-sectional design, which does not enable us to argue that there is a causal relationship. Despite these limitations, the strength of the study is the exhaustiveness of the data, as all eligible patients (expect for three) admitted during the study period were systematically screened. Well-designed intervention studies are now needed to better characterize the possible implications of AUD stigmatization after a suicide attempt and to provide effective strategies to improve AUD consideration in self-poisoning patients.

## Conclusion

Our study confirms previous data, which underline the lack of consideration of AUD co-morbidity in suicide attempters. It also underlines differences in how the condition is considered compared to depression, even though both psychiatric disorders are associated with a similar increased risk of suicide. Moreover, there are specific concerns about the efficacy of outpatient care for AUD patients, given that the AUD population is known to be less compliant with outpatient appointments. In the context of a need to prevent repeat suicide attempts, our results demonstrate that the issue of AUD and suicide must be specifically addressed.
